# Reacting to Perceived Overqualification: Uniting Strain-Based and Self-Regulatory Adjustment Reactions and the Moderating Role of Formal Work Arrangements

**DOI:** 10.1007/s10869-022-09870-8

**Published:** 2023-01-20

**Authors:** Maike E. Debus, Barbara Körner, Mo Wang, Martin Kleinmann

**Affiliations:** 1 University of Neuchâtel, Neuchâtel, Switzerland; 2 Leeds University Business School, Leeds, UK; 3 University of Florida, Gainesville, FL 32611 USA; 4 University of Zurich, Zurich, Switzerland

**Keywords:** Perceived overqualification, Performance, Person-environment fit, Job crafting

## Abstract

Thus far, research on perceived overqualification has focused on either maladaptive, strain-based versus more adaptive, self-regulatory reactions in isolation. Following person-environment fit theory, we seek to advance this one-sided focus by uniting both types of adjustment reactions and to consider their implications for perceived person-job fit, and performance and wellbeing outcomes. In line with theory, we also examine contextual boundary conditions in the form of indicators of formal work arrangements (i.e., permanent vs. temporary employment contract and job tenure). Utilizing three-wave data from 453 employees, we found that perceived overqualification indirectly and sequentially related to decreases in task performance, organizational citizenship behavior and job satisfaction via anger toward employment situation and lower levels of perceived person-job fit—thus reflecting the strain-based pathway. For the self-regulatory pathway, findings did not align with our initial proposition that the positive relationship between perceived overqualification and work organization (a form of structural job crafting whereby employees improve their work processes) would be weaker among temporary employees and those with longer tenure. Instead, having a temporary employment contract or having longer job tenure resulted in a negative relationship between perceived overqualification and work organization, which further contributed to a decrease in performance and satisfaction via lower levels of perceived person-job fit. Our study highlights the demotivating role of a temporary employment contract and long job tenure for overqualified employees to reorganize their work. In discussing our findings, we point to the importance of job stage and develop recommendations for managing overqualified employees.

Perceived overqualification, whereby employees feel they are in positions that do not fully utilize their skills and qualifications, has become a hot topic in the management and industrial-organizational psychology literature over the past decades (e.g., Erdogan & Bauer, 2021). One of the most commonly used theories to understand perceived overqualification and its outcomes is person-environment (P-E) fit theory (e.g., Edwards, 2008; Jansen & Kristof-Brown, 2006). According to this theory, perceived overqualification denotes a type of person-job misfit, whereby employees see a mismatch between their abilities, qualifications and needs and what their jobs demand from them and provide them in terms of responsibility and challenge (e.g., Edwards et al., 2006; Harari et al., 2017). P-E fit theory further proposes that when individuals encounter such situations of misfit, “two sets” (Edwards et al., 1998, p. 32) of simultaneously occurring adjustment reactions can result. The first type of adjustment reaction is a maladaptive, strain-based reaction. So far, the literature on perceived overqualification has mainly focused on this type of reaction, showing that employees who perceive themselves to be overqualified experience higher levels of strain and related detrimental outcomes (for meta-analytic evidence, see Harari et al., 2017).

Yet, according to P-E fit theory’s second type of adjustment reaction, employees can also engage in adaptive, self-regulatory behavior in order “to resolve P-E misfit” (Edwards et al., 1998, p. 32; see also French et al., 1974). Thus, employees may not passively endure misfit, but can actively shape the scope of their work to better match their abilities and needs (Follmer et al., 2018; Yu, 2013). Thus far, this alternative set of self-regulatory reactions to misfit has been largely overlooked not only in the literature on perceived overqualification, but also more generally in the P-E fit literature (for an exception see Follmer et al., 2018). Although there are some initial studies linking perceived overqualification with more active behaviors (e.g., proactive behavior, task crafting, Lin et al., 2017; Zhang et al., 2016, 2021), these studies have solely adopted a self-regulatory or job crafting theory lens. Thus, no study has so far simultaneously considered both strain-based and self-regulatory reactions and the outcomes that may result from misfit using a P-E fit lens (Edwards et al., 1998). Indeed, in their recent review of the perceived overqualification literature, Erdogan and Bauer (2021) pointed to earlier research’s one-sided focus on either maladaptive, strain-based vs. more adaptive reactions.

Because focusing on only one set of reactions may result in a rather fragmented understanding of the psychological processes triggered by perceived overqualification, we explicitly unite both pathways in line with P-E fit theory’s rationale in our research. We thus aim to achieve a more holistic understanding of the qualitatively different, yet co-occurring reactions that overqualified employees can demonstrate. In the present research, we examine—based on P-E fit theory—whether perceived overqualification simultaneously elicits a *strain-based pathway* and a *self-regulatory pathway* that have countervailing effects. We focus on *anger toward employment situation* as a typical strain indicator, and on *work organization* as an indicator of self-regulatory behavior in line with P-E fit theory (Follmer et al., 2018; French et al., 1982; Yu, 2013). Anger denotes a distinct affective reaction that is characterized by high negative arousal, displeasure and antagonism (e.g., Berkowitz & Harmon-Jones, 2004; Lindquist & Barrett, 2008). It occurs when employees’ goals, such as utilizing one’s competencies and receiving adequate recognition in the case of overqualified employees (Liu et al., 2015), are blocked (Berkowitz & Harmon-Jones, 2004). Central to the affective state of anger is the fact that is has an identifiable source or target (e.g., Basch & Fisher, 2000; Berkowitz & Harmon-Jones, 2004; Grandey et al., 2002; Lemerise & Dodge, 2008). In the present study, we focus on anger *toward employment situation* as a type of workplace anger (Fitness, 2000; Glomb, 2002) in order to contextually match this affective reaction with its proposed source (i.e., perceived overqualification as a stressor originating from the work situation; see Liu et al., 2015). Work organization is a type of job crafting whereby employees design “systems and strategies” to innovate and customize processes (Bruning & Campion, 2018, p. 509), thereby contributing to the maximization of their capabilities. This behavior should be highly relevant for overqualified employees as it allows them to transfer their previous experiences and competencies to their current job (Edwards et al., 1998; French et al., 1974; Yu, 2013). Accordingly, we first propose that perceived overqualification will be *negatively* related to two types of performance (i.e., task performance and organizational citizenship behavior towards the organization (OCBO)) as well as job satisfaction via higher levels of anger toward employment situation in the first step, which then contributes to lower levels of perceived person-job fit perceptions in the second step. Second, we propose that perceived overqualification will be *positively* related to the two performance outcomes and job satisfaction via higher levels of work organization in the first step, which then contributes to higher levels of person-job fit perceptions in the second step. Hence, in line with P-E fit theory (e.g., Edwards et al., 1998; Yu, 2013), we assume that changes in person-job fit perceptions channel the effects of the strain-based and the self-regulatory pathways on changes in job performance and job satisfaction.

Moreover, P-E fit theory suggests that the relative strength of the two simultaneously occurring pathways can be affected by “environmental resources and constraints” (Edwards et al., 1998, p. 32; see also Yu, 2013). Based on this notion, we argue that the strength of these pathways may depend on an employee’s *type of employment contract* (permanent employment contract vs. temporary contract) and their *job tenure*. Both contextual factors are key indicators of more formal work arrangements that can provide an important frame of reference for overqualified employees to interpret their work situation and to shape their strain- and self-regulatory reactions (Edwards et al., 1998). We specifically focus on type of contract and job tenure in the present study because these variables represent a type of “bond” that employees have with their organization and their job (e.g., De Witte & Näswall, 2003; Katz, 1980), respectively—thus bearing high significance for how overqualified employees react to their situation of misfit. The idea of contextual boundary conditions within P-E fit theory goes back to Lewin’s (1935; see also Pervin, 1989) field theory that stresses people’s reactions and behaviors to be co-determined by the context in which they are situated (see also Yu, 2013). Whereas previous research has examined several contextual boundary conditions of the perceived overqualification-outcome link, these studies have tended to narrowly focus on job design and organizational factors (e.g., autonomy, organizational prestige; for an overview, see Erdogan & Bauer, 2021). Thus, not much is known about whether formal characteristics of the work situation in which employees are embedded, such as the type of employment contract and job tenure, can shape reactions to perceived overqualification. Fig. 1 summarizes our hypothesized model.

**Fig. 1:**
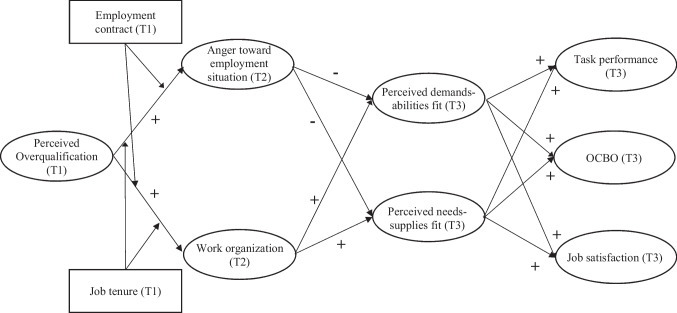
Conceptual model. T1/T2/T3 = first/second/third measurement time point

Our research makes several contributions to the literature. First, we apply P-E fit theory (e.g., Edwards et al., 1998) as an overarching theoretical framework to better understand differing employee reactions to perceived overqualification. Although related constructs have been separately tested in earlier research (e.g., Liu et al., 2015; Zhang et al., 2016), we integrate different adjustment reactions in line with P-E fit theory in one model and demonstrate their implications on performance and job satisfaction as two key types of outcomes that relate to individual contributions to the organization and to the individual person’s wellbeing (Edwards & Shipp, 2007), respectively. In doing so, we seek to advance the literature by arriving at a parsimonious, yet integrative framework that can better predict adjustment reactions to perceived overqualification—thus leading to a more comprehensive understanding of perceived overqualification and its outcomes. Second, by examining whether person-job fit perceptions channel the effects of the strain-based and the self-regulatory pathways on changes in job performance and job satisfaction, we contribute to the literature by testing a more dynamic feature of P-E fit theory. Despite the fact that P-E fit theory suggests that both strains as well as self-regulatory adjustment reactions provide feedback to person-job fit perceptions (e.g., Edwards et al., 1998; Yu, 2009), this mechanism has rarely been examined in the literature.

Third, our study contributes to a further theoretical fine-tuning of our predictions by incorporating P-E fit theory’s notion of contextual boundary conditions that shape the relative strength of each of the proposed paths (e.g., Edwards et al., 1998). While the role of such boundary conditions has been explicitly theorized, empirically, this aspect remains “a relatively unexplored research area” (Devloo et al., 2011, p. 26, for an exception, see Simmering et al., 2003). By theorizing and examining the moderating role of type of employment contract and job tenure, we not only examine a rather neglected aspect of the theory, but increase our understanding of the processes that shape the relative strength of the two adjustment reactions to perceived overqualification (see Devloo et al., 2011). In doing so, we also seek to meaningfully extend previously examined types of moderators for the effects of perceived overqualification. Research thus far has identified a broad range of crucial moderators, relating to personal, relational, job-related, and organizational characteristics (Erdogan & Bauer, 2021). By focusing on employment contract and job tenure, we enrich the current literature with more objective factors that reflect the formal work context in which employees are situated. Finally, considering these indicators of formal work arrangements is also highly relevant from a practical perspective, as it can provide important information to managers and organizations on how to prevent maladaptive reactions and foster adaptive ones to perceived overqualification.

## Theoretical Review and Hypotheses Development

### Perceived Overqualification in the Lens of Person-Environment Fit Theory

P-E fit theory suggests that when individuals perceive misfit, such as perceived overqualification, there are two different types of reactions that can occur simultaneously (Edwards et al., 1998; Follmer et al., 2018). First, individuals may react with strains, defined as deviations from normal functioning, which result in further detrimental outcomes. In the literature on perceived overqualification, strain reactions and the manifold associated detrimental outcomes (e.g., impaired job attitudes and well-being) are by far the most intensively researched type of adjustment in the context of P-E fit theory (e.g., Harari et al., 2017). Second, P-E fit theory proposes that individuals may also invest effort in order to reduce their situation of misfit, such as by engaging in self-regulatory behaviors (e.g., work organization)—thus possibly leading to more favorable and functional outcomes (Edwards et al., 1998; see also Kooij et al., 2020). Indeed, in a qualitative study, Follmer et al. (2018) shed initial light on this issue. The authors demonstrated that individuals oftentimes engage in actions whereby they try to resolve their misfit at work. The authors thus concluded that “the range of strategies used to address misfit is much more complex” (p. 459) than had previously been thought when the focus was only on strain reactions to misfit (see also French et al., 1974; Wheeler et al., 2005; Yu, 2013).

Perceived person-job fit, the second-stage mediator in our conceptual model, refers to the congruency that employees perceive between their own characteristics and those of their job; such perceptions tend to be idiosyncratic and holistic judgments (Kristof, 1996; Kristof-Brown & Billsberry, 2013). We will consider both *perceived demands-abilities fit* and *perceived needs-supplies fit* as the two broad classes of perceived person-job fit in line with P-E fit theory (Edwards et al., 1998; Yu, 2009). Whereas perceived demands-abilities fit refers to a person’s perceived compatibility between formal job requirements/job tasks and their knowledge, skills, and abilities (KSAs), perceived needs-supplies fit refers to the degree to which individuals perceive that the job fulfills their goals, values, and desires (Beier et al., 2020; Edwards, 1991; Kristof, 1996). Person-job fit is thus conceptually different than other P-E fit dimensions (e.g., person-organization fit, person-vocation fit) due to its explicit focus on the job and the tasks that it comprises (e.g., Jansen & Kristof-Brown, 2006; Kristof, 1996).

In terms of outcomes, we consider task performance, OCBO, and job satisfaction. Whereas task performance refers to the extent to which employees engage in activities that are formally identified in their job description, OCBO denotes a type of contextual performance, referring to any voluntary behaviors that are not formally required of employees, but that aim to benefit the organization as a whole (e.g., Borman & Motowidlo, 1993). We chose these two performance outcomes due to their high relevance from an organizational point of view; that is, whereas task performance refers to fulfilling one’s job description as it was defined by the company, OCBO targets voluntary behavior that explicitly aims to benefit the organization. Moreover, we chose OCBO because the target of this behavior (i.e., the organization) likely aligns with the primary target of overqualified employees’ attributions. In other words, employees who perceive themselves to be overqualified likely first and foremost attribute this situation to the organization (e.g., Liu et al., 2015) that is not able to provide them with a job that better matches their qualifications. Finally, job satisfaction—“a pleasurable or positive emotional state resulting from the appraisal of one’s job or job experiences” (Locke, 1976, p. 1304)—constitutes the most central subjective well-being indicator in the work context (e.g., Judge & Klinger, 2007). To underscore the generalizability of our assumptions, we consider both performance outcomes as well as work-related wellbeing outcomes. Indeed, both types of outcomes represent key outcomes that have been theorized in the context of P-E fit theory (e.g., Edwards & Shipp, 2007; Kristof-Brown et al., 2005).

### Sequential Mediation of the Strain-Based and Self-Regulatory Pathways via Perceived Person-Job Fit on Job Performance

In the present research, we first argue, based on P-E fit theory, that perceived overqualification will elicit a strain-based pathway, such that higher levels of anger toward employment situation and lower levels of perceived person-job fit sequentially mediate the relationship between perceived overqualification and decreases in both task performance, OCBO, as well as job satisfaction. Generally, anger is a discrete affective reaction that arises when individuals “are kept from attaining an important goal by an external agent’s improper action” (Berkowitz & Harmon-Jones, 2004, p. 109). Individuals can experience anger both as a short-lived, intense emotional reaction to a specific event, or in the form of a longer lasting mood state (e.g., Harmon-Jones & Harmon-Jones, 2016). Because we examine anger as a reaction to perceived overqualification as a relatively enduring work stressor in line with the extant literature (Erdogan & Bauer, 2021), we consider anger to be a mood state in the present study (Andel et al., 2022; Liu et al., 2015). Due to the central role of the target or source of anger experiences, anger *toward employment situation* should be most relevant when examining reactions to perceived overqualification (Liu et al., 2015) as an employment situation characteristic. In utilizing a contextualized form of anger, we follow earlier approaches in the literature (Chen & Spector, 1992; Liu et al., 2015; Spencer & Rupp, 2009); moreover, based on research on item contextualization effects (Hunthausen et al., 2003; Lievens et al., 2008), we assume high predictive relevance of perceived overqualification for anger toward employment situation due to the contextual match of both constructs. More precisely, and relating to the above notion of goal obstruction central to the experience anger, for employees who perceive themselves to be overqualified one of the major goals in the work context—demonstrating and utilizing their competencies (e.g., Deci & Ryan, 1985; Hackman & Oldham, 1976)—is obstructed. Likewise, these employees feel deprived of receiving adequate recognition for qualifications that they have (Liu et al., 2015). We thus assume that perceived overqualification should be predictive of anger toward employment situation, precisely because it is the employment situation with their tasks and responsibilities that does not allow overqualified employees to adequately utilize and exploit their skills and qualifications (for evidence, see Andel et al., 2022; Liu et al., 2015; Luksyte et al., 2022).

Next, considering P-E fit theory’s more recent propositions about the impact of affective experiences for perceptions of (mis)fit (Edwards et al., 2006; Yu, 2009), anger toward employment situation likely contributes to lower levels of perceived demands-abilities and needs-supplies fit. For example, Yu (2009) proposed an expanded model of P-E fit whereby he drew from theories of emotion processes like the affect-as-information framework (e.g., Clore et al., 2001). The framework suggests that individuals use their affective states as information to further guide their perceptions, judgments and decision making. In the present context, this rationale suggests that anger would make the situation of low perceived person-job fit more salient to overqualified employees. More concretely, the anger associated with perceived overqualification may increase employees’ feelings that their jobs provide a poor fit to the manifold qualifications and abilities that they bring with them (demands-abilities fit) and can only poorly satisfy their needs for competence and responsibility (needs-supplies fit).

Finally, the resulting low perceived person-job fit may contribute to a decrease in task performance, OCBO, and job satisfaction. First, for the two performance outcomes, social exchange theory (Blau, 1964) suggests that if employees experience poor fit with their demands and the fulfillment of their needs, their commitment to their organization is reduced. This low commitment, in turn, makes employees less willing to invest effort into high task performance and to perform at their maximum capability levels; likewise, employees’ low levels of commitment will make them less likely to reciprocate by investing more time to and energy into behaviors that go beyond formal expectations, such as OCBO in the present case (Brief & Motowidlo, 1986; Kristof-Brown et al., 2005; LePine et al., 2002). Second, for job satisfaction, when employees experience poor demands-abilities fit, this likely hampers their sense of competence, mastery, and adequate skill utilization (Edwards & Shipp, 2007; Feather, 1991)—thus impairing their job-related wellbeing, which manifests in lower levels of job satisfaction (Edwards & Shipp, 2007; Kristof-Brown et al., 2005). Moreover, for perceived needs-supplies fit, theorizing on job satisfaction suggests that individuals engage in a comparison process whereby they compare valued or desired amounts of job features with what they actually have (e.g., Dawis & Lofquist, 1984; Locke, 1969). This reasoning suggests that if employees experience poor needs-supplies fit, the comparison process results in a discrepancy indicating inadequate needs fulfilment which makes employees more likely to be dissatisfied with their job (Edwards & Shipp, 2007, for meta-analytic evidence about the relationship between perceived fit as well as performance and job satisfaction see Kristof-Brown et al., 2005). We thus propose:

- *Hypothesis 1*: Higher levels of anger toward employment situation and lower levels of perceived person-job fit sequentially mediate the relationships between perceived overqualification and decreases in (a) task performance, (b) OCBO, and (c) job satisfaction.

Next, we argue, based on P-E fit theory, that perceived overqualification will elicit a self-regulatory pathway, such that higher levels of work organization and higher levels of perceived person-job fit sequentially mediate the relationship between perceived overqualification and increases in task performance, OCBO, and job satisfaction. Work organization is a form of structural job crafting whereby employees actively change the boundaries of their work tasks and the procedures of their work (Bruning & Campion, 2018; Wrzesniewski & Dutton, 2001). When engaging in this behavior, employees aim to improve given structures and procedures (Bruning & Campion, 2018) by actively and autonomously designing systems and (behavioral) strategies that help them to better organize the tangible elements of work. In doing so, employees contribute to process innovation and customization of their work, and a maximization of their capabilities (Bruning & Campion, 2018). We focus on work organization in the present context because it matches P-E fit theory’s underlying theme of the self-regulatory pathway whereby employees engage in activities aimed at improving their situation and resolving states of P-E misfit (Edwards et al., 1998). Due to their various past experiences, overqualified employees would be expected to engage in work organization, because these employees are more likely to discover opportunities where given procedures and strategies can be improved, such that they can make better use of their abilities and may better satisfy their need for competence. In fact, this reasoning is also echoed in Edwards et al.’s (1998) notion of ‘carryover’, meaning that employees can transfer their excess abilities to other tasks in order to deal with this situation of misfit.

Following this argument, and in line with the theoretical rationale of P-E fit theory’s self-regulatory pathway (Edwards et al., 1998), work organization should be positively related to increases in task performance, OCBO and job satisfaction precisely because this behavior should lead to higher levels of perceived demands-abilities and needs-supplies fit (Kristof-Brown et al., 2005). As noted before, self-regulatory behaviors within the context of P-E fit theory have been explicitly theorized to improve the fit with one’s environment (Edwards et al., 1998; Yu, 2013), such as perceived person-job fit in the present context (for preliminary evidence see Tims et al., 2016). In line with our earlier theorizing, the resulting higher levels of person-job fit will contribute to an increase in task performance, OCBO, and job satisfaction. This is because high levels of person-job fit make employees experience more organizational commitment, thus making them more willing to demonstrate high effort at work, which contributes to high task performance, and to invest resources into organizationally beneficial behaviors, such as OCBO (Kristof-Brown et al., 2005). Similarly, higher levels of perceived person-job fit make employees more likely to experience a sense of competence and an adequate needs fulfilment which would make them more satisfied with their jobs (Edwards & Shipp, 2007; Kristof-Brown et al., 2005). We thus propose:

- *Hypothesis 2*: Higher levels of work organization and person-job fit sequentially mediate the relationships between perceived overqualification and increases in (a) task performance, (b) OCBO, and (c) job satisfaction.

### Type of Employment Contract and Job Tenure as Contextual Moderators

Based on P-E fit theory’s notion of contextual boundary conditions (Edwards et al., 1998; Yu, 2013), we further propose that indicators of formal work arrangements are crucial factors that shape how employees who perceive themselves to be overqualified will interpret and judge their situation. First, we expect a permanent (as opposed to a temporary) employment contract to weaken the relationship between perceived overqualification and anger toward employment situation and to strengthen the relationship between perceived overqualification and work organization. Whereas permanent contracts are open-ended in their duration, temporary contracts have a set end date. Temporary employment is similar to contingent employment, a term commonly used in the USA and Canada (Kalleberg, 2000). In the literature, temporary contracts are viewed as a type of “precarious” employment (Letourneux, 1998), “non-standard” employment (OECD, 2019), or “atypical” work (Giesecke & Gross, 2004); temporary contracts are therefore considered an objective form of job insecurity (Pearce, 1998), referring to the likelihood that an individual might lose their job in the future. Due to manifold economic pressures, many companies nowadays hire their employees on a fixed-term basis (e.g., OECD, 2019). This is evident by the increased amount of temporary contracts over the past decades, and the likelihood that this trend will continue to rise due to companies’ pressures to adapt their workforce in competitive markets (e.g., OECD, 2021). In the context of P-E fit theory, type of contract has indeed previously been posited as a moderating factor in shaping reactions to P-E misfit (Sekiguchi, 2007), yet strong empirical evidence is still needed.

With regard to effects on anger toward employment situation, employees may view their situation of overqualification as somewhat less detrimental if they hold a permanent contract because it signifies security and a long-term perspective with their current employer. Thus, even though the situation of perceived overqualification constitutes a situation of misfit and goal-blockage, employees with a permanent contract nevertheless have a long-term relationship with their employer which may increase their self-worth and self-esteem (e.g., Hughes & Palmer, 2007). This may help overqualified individuals to better tolerate their situation. In contrast, employees on a temporary contract may find themselves in a situation where poor fit is coupled with low job security, which would make them even more likely to feel that their work-related goals are blocked. Taken together, it thus follows that the positive relationship between perceived overqualification and anger toward employment situation will be weaker among employees on a permanent contract and stronger among employees on a temporary contract.

- *Hypothesis 3a*: Employment contract moderates the positive relationship between perceived overqualification and anger toward employment situation, such that the relationship will be weaker when the contract is permanent (vs. a temporary contract).

Further, employees who perceive themselves to be overqualified should be more likely to engage in work organization if they hold a permanent contract as opposed to a temporary contract. As noted before, having a permanent contract signals job security and longevity within one’s company (e.g., De Witte & Näswall, 2003). Under this condition, overqualified employees will be particularly likely to improve and optimize their jobs, as doing so may help them to customize their job according to their qualifications and needs on a long-term basis. They are also more likely to be rewarded by taking this action in a long run. Conversely, employees who are employed on a temporary contract are with their company on a time-limited basis (Seijts, 1998). As such, investing time and energy into organizing and optimizing processes and strategies might not be worth the effort for them because these individuals have to leave the organization at a set date. We thus propose:

- *Hypothesis 3b*: Employment contract moderates the positive relationship between perceived overqualification and work organization, such that the relationship will be stronger when the contract is permanent (vs. a temporary contract).

Second, job tenure refers to the “length of time in one position” (Ng & Feldman, 2013, p. 306). In many European countries, tenure is a key indicator of formal work arrangements (e.g., OECD, 2022b). As an example, in Germany, where we collected our data, employees with longer tenure are protected by the Civil Code with a longer period of minimum notice in the case of dismissal (Eurofound, 2021), and they usually receive more organizational benefits such as more vacation days and loyalty bonuses by their employer (Arbeitsrechte.de, 2021; Hausen, n.d.). Despite these positive aspects associated with tenure, we argue that job tenure strengthens the relationship between perceived overqualification and anger toward employment situation, and weakens the relationship between perceived overqualification and work organization. More precisely, theorizing on different job stages (Murphy, 1989) highlights that employees first enter a “transition stage,” whereby they get acquainted with their new jobs and its tasks, and are ambitious about their work goals. After this period, employees enter a “maintenance stage” in which task routines are established, work goals become less ambitious, and motivation declines (see also Katz, 1980). Related to this, the job and career plateauing literature suggests that with longer job tenure, that is, when employees are in the maintenance stage, they oftentimes experience being stuck in their jobs and experience an overall sense of staleness toward their job (e.g., Elsass & Ralston, 1989; Yang et al., 2019). This feeling of being stuck occurs because employees experience a lack of growth, challenges and/or responsibilities, and a discrepancy between their career goals/aspirations and their current situation (e.g., Allen et al., 1999). In their conceptual paper, Elsass and Ralston (1989) argue that a major characteristic of this situation is the strain that employees experience (for empirical evidence see McCleese et al., 2007; see also Yang et al., 2019), and that strain can intensify with longer job tenure. We thus propose that with increasing job tenure, that is, the longer this situation lasts, overqualified employees will develop a stronger experience of strain and become more aware of the fact that their goals of adequately utilizing their qualifications and demonstrating their competencies are blocked. Thus, the relationship between perceived overqualification and anger toward employment situation will be stronger with increasing tenure.

- *Hypothesis 4a*: Job tenure moderates the positive relationship between perceived overqualification and anger toward employment situation, such that the relationship will be stronger when job tenure is higher.

Further, we propose that employees who perceive themselves to be overqualified will be less likely to engage in work organization with increasing job tenure. Murphy’s (1989) job stage model as well as job design theory (Hackman, 1978) suggest that with increasing job tenure, employees’ motivation and enthusiasm decline (see also Katz', 1980, job experience model). Indeed, Ng and Feldman (2013) found that performance gains due to human capital acquisition among employees with longer job tenure were offset by losses of intrinsic motivation. Thus, the relationship between perceived overqualification and work organization should be weaker for employees with longer job tenure, but stronger for employees with shorter job tenure. As noted previously, work organization refers to the active design of systems and strategies in order to “get more resource values out of the set of tasks one currently has” (Bruning & Campion, 2018, p. 509). Overqualified employees with longer job tenure are likely to be highly routinized in their work strategies and procedures (e.g., Murphy, 1989), such that they might be less attentive and aware as to where work organization behaviors might be beneficial to them, as well as less willing to execute them. In contrast, overqualified employees with shorter job tenure may still bring more willingness for change as well as higher awareness with them as to where work organization behaviors may result in beneficial resource gains for them. This higher awareness for opportunities to engage in work organization likely results from their past job experiences that would be still more salient to employees with shorter job tenure. Indirect support for our proposition comes from a study by Tims et al. (2013) who found a direct negative effect of tenure on various job crafting behaviors.

- *Hypothesis 4b*: Job tenure moderates the positive relationship between perceived overqualification and work organization, such that the relationship will be weaker when job tenure is higher.

## Method

### Sample and Procedure

Data were collected at three time points with a one-month lag between each wave through a market research company (for a similar approach see Batinic et al., 2010; Debus et al., 2019).^1^ In exchange for their participation, participants received bonus points they could later redeem at different online stores or donate to a charity organization. We chose the 1-month time lag as per Dormann and Griffin’s (2015) recommendation on using “shortitudinal” designs and because earlier research (e.g., Tims et al., 2013) also demonstrated a 1-month lag to be appropriate for effects of job crafting to become visible. Participants qualified for study participation if they provided their consent to participate, were currently working for pay, were 18 years or older, and were not students, apprentices and/or doing an internship, had not resigned from their current job or been dismissed, and had been working during the preceding four weeks. We assessed the predictor, moderators and control variables at T1, first stage mediators at T2, and second stage mediators and outcomes at T3 (see below for more detailed information). At T2 and T3 we also assessed whether participants still held the same job. If this was not the case, they were screened out.

In total, 1234 participants provided their consent to take part in the survey at T1. Of those, we excluded 248 who did not meet the abovementioned selection criteria; we further excluded 84 participants who failed the attention check in the middle of the survey, and 32 participants who did not provide complete surveys. Moreover, we excluded 56 participants who completed the survey within a too short time interval (i.e., below half of the median response time of 8.28 min) in line with recommendations by the European Society for Opinion and Market Research (ESOMAR, Respondi, n.d.), as well as two double entries (where we always used the later entry). This resulted in a total of 812 participants who provided usable data at T1 and who were invited to complete the T2 survey. During the course of the study, 359 participants dropped out due to non-response or because they did not pass quality checks at T2 or T3 and where thus excluded from the final sample.

The final sample consisted of 453 participants, leading to a response rate of 55.79%. To examine whether systematic differences existed between employees in the final sample (*N* = 453) vs. employees who dropped out during the course of the study (*N* = 359), we conducted chi-square and *t-*tests on demographic variables as well as study variables (i.e., predictor and moderator variables). Results showed no differences between the two groups in terms of education and perceived overqualification. Participants in the final sample were slightly older, were more often employed on a permanent basis, and had slightly higher job tenure.^2^ In the final sample, 48.34% of participants were female. In terms of education, 5.52% fulfilled the mandatory years at school, 30.68% had a vocational training, 20.97% had a university entrance diploma, 19.87% had a technical college or master craftsman’s diploma, and 22.96% had a university degree.

### Measures

We followed Brislin’s (1970) translation/back-translation procedure to translate those measures for which there was no German version available (i.e., anger toward employment situation and work organization). All variables, unless indicated otherwise below, were measured on a 7-point rating scale ranging from (1) *strongly disagree* to (7) *strongly agree*. For work organization and the two performance measures, employees were instructed to respond to the items with regards to their behavior during the preceding 4 weeks (i.e., time since the last survey). Cronbach’s alphas for all measures are displayed in the main diagonal of Table 1.

**Table 1:** Means, standard deviations, and zero-order correlations among study variables

Variables		*M*	*SD*	1	2	3	4	5	6	7	8	9	10	11	12	13	14	15	16	17	18
1	Age (T1)	45.04	11.95	–																	
2	Education (T1)	2.24	1.26	− .08	–																
3	Neuroticism (T1)	3.11	1.33	− .29**	− .01	(.88)															
4	Employment contract (T1)	–	–	− .11*	.03	.02	–														
5	Job tenure (T1)	8.78	8.46	.50**	− .14**	− .18**	− .12 *	–													
6	Perceived overqualification (T1)	3.87	1.33	.00	− .01	.22**	.03	− .12**	(.91)												
7	Demands-abilities fit (T1)	5.93	0.91	.22**	.00	− .42**	− .03	.18**	− .22**	(.84)											
8	Needs-supplies fit (T1)	5.41	1.28	.08	.03	− .43**	.03	.13**	− .38**	.63**	(.94)										
9	Task performance (T1)	6.07	0.85	.26**	− .10*	− .29**	− .02	.23**	− .04	.54**	.33**	(.89)									
10	OCBO (T1)	4.52	1.39	.04	.15**	− .25**	.01	.03	− .16**	.21**	.43**	.14**	(.87)								
11	Job satisfaction (T1)	5.48	1.41	.15**	− .04	− .42**	.02	.15**	− .36**	.55**	.80**	.40**	.34**	(.91)							
12	Anger toward employment situation (T2)	2.53	1.48	− .16**	.05	.40**	− .02	− .16**	.33**	− .42**	− .53**	− .32**	− .19**	− .69**	(.91)						
13	Work organization (T2)	4.36	1.43	− .05	.04	− .14**	.00	.00	− .13**	.16**	.26**	.08	.47**	.20**	− .12*	(.91)					
14	Demands-abilities fit (T3)	6.04	0.88	.23**	− .04	− .39**	− .01	.25**	− .17**	.67**	.50**	.50**	.20**	.48**	− .41**	.16**	(.81)				
15	Needs-supplies fit (T3)	5.50	1.25	.13**	− .03	− .42**	.02	.22**	− .41**	.48**	.76**	.32**	.40**	.74**	− .55**	.29**	.59**	(.95)			
16	Task performance (T3)	6.17	0.79	.29**	− .11*	− .32**	− .05	.25**	− .04	.45**	.31**	.65**	.15**	.37**	− .31**	.12*	.58**	.37**	(.85)		
17	OCBO (T3)	4.56	1.38	.03	.12**	− .18**	.01	.00	− .19**	.17**	.39**	.10*	.73**	.33**	− .20**	.46**	.20**	.43**	.16**	(.87)	
18	Job satisfaction (T3)	5.53	1.34	.12*	− .09	− .41**	.04	.16**	− .34**	.45**	.69**	.37**	.31**	.82**	− .69**	.23**	.50**	.80**	.40**	.36**	(.90)

*N* = 453. T1/T2/T3 = first/second/third measurement time point. Alpha reliabilities appear in the parentheses along the diagonal. For education: mandatory years of education = 0; vocational training = 1; university entrance diploma = 2; technical college or master craftsman’s diploma = 3; university degree = 4. For employment contract: permanent contract = 0; temporary contract = 1. Variables 3 and 6 to 18 were measured on a 7-point rating scale ranging from (1) strongly disagree to (7) strongly agree

^*^ *p* < .05; ** *p* < .01 (two-tailed)

#### Perceived Overqualification (Time 1 Survey)

Perceived overqualification was measured with the nine-item Scale of Perceived Overqualification by Maynard et al. (2006, e.g., “I have more abilities than I need in order to do my job.”).

#### Type of Contract (Time 1 Survey)

Employees’ type of contract was assessed with the following question: “What kind of contract do you have?” Participants could choose between “permanent contract” and “temporary contract” (see Debus et al., 2014).

#### Tenure (Time 1 Survey)

Employees’ job tenure was assessed with the following question: “Since when have you been working in this position? Please indicate your starting date.” Based on their answers, we then calculated the number of years that employees had been working in their current position.

#### Anger Toward Employment Situation (Time 2 Survey)

Anger toward employment situation was measured using the respective three-item measure by Liu et al. (2015, adapted from Chen & Spector, 1991; e.g., “I feel angry about the job assignment I received from my employer.”).

#### Work organization (Time 2 survey)

Work organization was measured with the respective four-item measure by Bruning and Campion (2018, e.g., “I created structure in my work processes.”).

#### Perceived Demands-Abilities Fit (Time 3 Survey)

Perceived demands-abilities fit was measured with four items from Luksyte et al. (2011, based on Cable & Judge, 1996, e.g., “My knowledge, skills, and abilities match the requirements of my job.”).

#### Perceived Needs-Supplies Fit (Time 3 Survey)

Perceived needs-supplies fit was measured with four items from Luksyte et al. (2011, based on Saks & Ashforth, 1997, e.g., “I feel that this job enables me to do the kind of work I want to do.”).

#### Task Performance (Time 3 Survey)

Task performance was measured using five items from Staufenbiel and Hartz (2000, e.g., “I fulfilled all the responsibilities required by my job.”), a German-language version of the scale by Williams and Anderson (1991). For the use of this same scale, see Ingold et al. (2015).

#### OCBO (Time 3 Survey)

OCBO was measured using five items from Staufenbiel and Hartz (2000, e.g., “I contribute innovative ideas for quality improvement.”). For the use of this same scale, see Lehmann-Willenbrock et al. (2013).

#### Job Satisfaction (Time 3 Survey)

Job satisfaction was measured using the three-item job satisfaction subscale from the Michigan Organizational Assessment Questionnaire (MOAQ-JSS, Cammann et al., 1983, e.g., “I am satisfied with my job.”).

#### Control Variables

In line with prior research in the field of perceived overqualification, we controlled for *age* and *education* (e.g., Deng et al., 2018; Zhang et al., 2016) because these variables are likely to affect our mediator and outcome variables (Spector & Brannick, 2011). In particular, it has been shown that older employees demonstrate higher levels of job satisfaction (Ng & Feldman, 2010) and extra-role performance (Ng & Feldman, 2008). Likewise, employees with higher levels of education have been demonstrated to show higher levels of perceived person-job fit (Ilies et al., 2019) and job performance (Ng & Feldman, 2009). All these variables were assessed in the Time 1 survey. We also included *neuroticism* (five-item scale by Rammstedt & John, 2005, e.g., “I get depressed easily.”) in the T1 survey as a control variable for T2 measures (i.e., first stage mediators) due to its intensively discussed role in the stress process. More precisely, we did so in order to control for spillover effects of negative emotionality due to perceived overqualification (e.g., Maynard et al., 2006). People high in neuroticism tend to appraise stimuli around them as more threatening, thus fostering more negative reactions (e.g., Oliver et al., 2010; Spector et al., 2000). In line with Becker (2005), all control variables did show significant correlations with either dependent variables or the mediators (see Table 1). Finally, to be able to model the change in perceived person-job fit, performance, and job satisfaction and to account for the fact that these measures were assessed at the same point in time, we also assessed all these measures at T1 and controlled for them when predicting the respective T3 measures.

## Results

### Preliminary Analysis

Means, standard deviations, and correlations of the study variables are displayed in Table 1. Results of CFA to examine the construct validity of all study measures are displayed in Table 2. In sum, CFA showed that the proposed 14-factor measurement model [see M_0_ in Table 2; i.e., whereby neuroticism, perceived overqualification (both T1), anger, work organization (both T2), demands-abilities fit, needs-supplies fit, task performance, OCBO, and job satisfaction (all T3) and their respective T1 baseline measures each represent distinct constructs] fit the data better than alternative models (i.e., M_1_ and M_2_, see Table 2). We thus used M_0_ for further tests of structural equation models.

**Table 2:** Comparison of fit of models

Overview of models^1^	*χ*^2^ (*df*)	*∆χ*^2^ (*df*)	CFI	TLI
1) M_0_ (fourteen-factor measurement model based on the item parcels and observed covariates employment contract and job tenure, i.e., employment contract and job tenure were set to correlate with all latent factors)	1586.663*** (784) SCF: 1.1311		.95	.93
2) M_1_ (three-factor model where T1, T2 and T3 constructs each loaded on one latent factor and observed variables employment contract and job tenure as covariates)	9172.700*** (894) SCF: 1.1779	11,296.998*** (110) ^a^	.44	.40
3) M_2_ (one factor for fit measures and performance measures at T1 and T3; all other constructs represented single factors, and observed variable employment contract and job tenure as covariates)	4069.134*** (838) SCF: 1.1479	3242.020*** (54) ^b^	.78	.75
4) M_3_ (partial mediation model)	2044.765*** (881) SCF: 1.1243		.92	.91
5) M_4_ (full mediation model)	2049.437*** (885) SCF: 1.1243	4.672 (4) ^a^	.92	.91
6) M_5_ (moderated mediation model)		15.861 ** (4) ^a c^		

*N* = 453. T1/T2/T3 = first/second/third measurement time point. We used item parceling to model the respective latent constructs. This was done to avoid exceeding the recommended parameter-to-sample-size ratio for estimation (1:5, Bentler & Chou, 1987), which would have been the case had all scale items been included as observed indicators in the respective models

^1^All constructs were parceled with the single-factor method (Little et al., 2002), except anger toward employment situation (measured with three items), which was not parceled. Employment contract and job tenure (i.e., moderators) were included in the CFAs to ensure that the subsequent structural equation models for hypotheses testing were nested in the measurement model

*df* degrees of freedom, *SCF* scaling correction factor for MLR, *CFI* comparative fit index, *TLI* Tucker-Lewis index

^a^Model was compared with the previous one

^b^Model was compared to M_0_

^c^For latent interaction models, we calculated model comparisons based on the raw, uncorrected difference between the two log-likelihood values of the full mediation model and the moderated mediation model (Maslowsky et al., 2015)

^*^p < .05; ** *p* < .01; *** *p* < .001

### Hypothesis Testing

We applied structural equation modelling to estimate the proposed hypotheses. As direct effects of perceived overqualification on perceived demands-abilities fit (γ =  − 0.011, *p* = 0.779), task performance (γ = 0.044, *p* = 0.053), OCBO (γ =  − 0.011, *p* = 0.782), and job satisfaction (γ = 0.037, *p* = 0.268) were not significant in a partial mediation model (M_3_), we selected the more parsimonious full mediation model (M_4_) to test hypotheses 1 and 2 (see Table 2 for the model comparison between M_3_ and M_4_). In this model (M_4_), anger and work organization (i.e., first stage mediators) were predicted by perceived overqualification, employment contract, job tenure, and the control variables (i.e., age, education and neuroticism); perceived needs-supplies fit and demands-abilities fit (i.e., second stage mediators) were predicted by employment contract, job tenure, the first stage mediators, the control variables (i.e., age and education), and, in the case of perceived needs-supplies fit, by perceived overqualification (this effect was significant in the partial mediation model). We also controlled for the baseline levels (T1) of perceived needs-supplies fit and demands-abilities fit, respectively. Finally, task performance, OCBO, and job satisfaction (i.e., outcomes) were predicted by employment contract, job tenure, second stage mediators, the control variables (i.e., age and education) as well as the baseline levels (T1) of task performance, OCBO, and job satisfaction. We used Monte Carlo simulations with 20,000 replications (Selig & Preacher, 2008) to obtain the 95% confidence intervals (CIs) to examine the sequential mediation effects proposed in hypotheses 1 and 2. Table 3 summarizes parameter estimates and corresponding CIs based on a full mediation model (M_4_). Hypothesis 1a (i.e., perceived overqualification → [ +] anger → [-] perceived fit → [-] task performance) was supported for perceived demands-abilities fit but not for perceived needs-supplies fit, because perceived needs-supplies fit was not related to changes in task performance (γ = 0.013, *p* = 0.713). Hypothesis 1b (i.e., perceived overqualification → [ +] anger → [-] perceived fit → [-] OCBO) was supported for perceived needs-supplies fit but not for perceived demands-abilities fit, because perceived demands-abilities fit was not related to changes in OCBO (γ = -0.005, *p* = 0.947). Hypothesis 1c (i.e., perceived overqualification → [ +] anger → [-] perceived fit → [-] job satisfaction was supported for perceived needs-supplies fit but not for perceived demands-abilities fit, because perceived demands-abilities fit was not related to changes in job satisfaction (γ =  − 0.014, *p* = 0.826). Hypothesis 2a (i.e., perceived overqualification → [ +] work organization → [ +] perceived fit → [ +] task performance), hypothesis 2b (i.e., perceived overqualification → [ +] work organization → [ +] perceived fit → [ +] OCBO), and hypothesis 2c (i.e., perceived overqualification → [ +] work organization → [ +] perceived fit → [ +] job satisfaction) were not supported because perceived overqualification was not related to work organization (γ =  − 0.103, *p* = 0.139).

**Table 3:** Unstandardized estimates and bias-corrected confidence intervals of indirect path coefficients of the full mediation model (M_4_) and the moderated mediation model (M_5_)

	Indirect effect	
Variables	Estimate	Bias-corrected 95% CI
Perceived overqualification → *anger toward employment situation* → demands-abilities fit → task performance		
Indirect effect (M_4_)	− .005	[− .011, − .001]
Moderator employment contract: conditional indirect effects (moderated mediation model, M_5_)		
Temporary contract (TC)	− .006	[− .018, .0004]
Permanent contract (PC)	− .005	[− .012, − .0004]
Difference between TC and PC conditions	− .001	[− .001, .006]
Moderator job tenure: conditional indirect effects (moderated mediation model, M_5_)		
High job tenure (HJT)	− .005	[− .011, − .001]
Low job tenure (LJT)	− .005	[− .013, − .0002]
Difference between HJT and LJT conditions	.000	[− .003, 003]
Perceived overqualification → *anger toward employment situation* → needs-supplies fit → task performance		
Indirect effect (M_4_)	− .001	[− .004, .003]
Moderator employment contract: conditional indirect effects (moderated mediation model, M_5_)		
Temporary contract (TC)	− .001	[− .007, .004]
Permanent contract (PC)	− .001	[− .004, .003]
Difference between TC and PC conditions	− .0002	[− .004, .002]
Moderator job tenure: conditional indirect effects (moderated mediation model, M_5_)		
High job tenure (HJT)	− .001	[− .004, .003]
Low job tenure (LJT)	− .001	[− .005, .003]
Difference between HJT and LJT conditions	.000	[− .001, .001]
Perceived overqualification → *anger toward employment situation* → demands-abilities fit → OCBO		
Indirect effect (M_4_)	.0001	[− .005, .005]
Moderator employment contract: conditional indirect effects (moderated mediation model, M_5_)		
Temporary contract (TC)	− .009	[− .002, .006]
Permanent contract (PC)	.002	[− .002, .007]
Difference between TC and PC conditions	.0001	[− .004, .004]
Moderator job tenure: conditional indirect effects (moderated mediation model, M_5_)		
High job tenure (HJT)	.0002	[− .005, .006]
Low job tenure (LJT)	.0002	[− .006, .006]
Difference between HJT and LJT conditions	.000	[− .002, .002]
Perceived overqualification → *anger toward employment situation* → needs-supplies fit → OCBO		
Indirect effect (M_4_)	− .010	[− .020, − .002]
Moderator employment contract: conditional indirect effects (moderated mediation model, M_5_)		
Temporary contract (TC)	− .012	[− .033, .001]
Permanent contract (PC)	− .009	[− .022, − .001]
Difference between TC and PC conditions	− .003	[− .019, .011]
Moderator job tenure: conditional indirect effects (moderated mediation model, M_5_)		
High job tenure (HJT)	− .009	[− .020, − .002]
Low job tenure (LJT)	− .009	[− .024, − .001]
Difference between HJT and LJT conditions	.000	[− .006, .006]

Perceived overqualification → *anger toward employment situation *→ demands-abilities fit → job satisfaction		
Indirect effect (M_4_)	.0004	[− .004, .004]
Moderator employment contract: conditional indirect effects (moderated mediation model, M_5_)		
Temporary contract (TC)	.001	[− .007, .006]
Permanent contract (PC)	.0004	[− .004, .004]
Difference between TC and PC conditions	.0001	[− .004, .003]
Moderator job tenure: conditional indirect effects (moderated mediation model, M_5_)		
High job tenure (HJT)	.0004	[− .004, .004]
Low job tenure (LJT)	.0004	[− .004, .004]
Difference between HJT and LJT conditions	.000	[− .001, .001]
Perceived overqualification → *anger toward employment situation *→ needs-supplies fit → job satisfaction		
Indirect effect (M_4_)	− .020	[− .039, − .007]
Moderator employment contract: conditional indirect effects (moderated mediation model, M_5_)		
Temporary contract (TC)	− .025	[− .066, .001]
Permanent contract (PC)	− .019	[− .042, − .005]
Difference between TC and PC conditions	− .006	[− .038, .023]
Moderator job tenure: conditional indirect effects (moderated mediation model, M_5_)		
High job tenure (HJT)	− .019	[− .038, − .007]
Low job tenure (LJT)	− .019	[− .047, − .003]
Difference between HJT and LJT conditions	.000	[− .012, .012]
Perceived overqualification → *work organization *→ demands-abilities fit → task performance		
Indirect effect (M_4_)	− .001	[− .004, .0004]
Moderator employment contract: conditional indirect effects (moderated mediation model, M_5_)		
Temporary contract (TC)	− .007	[− .017, − .0004]
Permanent contract (PC)	.001	[− .001, .005]
Difference between TC and PC conditions	− .008	[− .020, − .001]
Moderator job tenure: conditional indirect effects (moderated mediation model, M_5_)		
High job tenure (HJT)	− .001	[− .003, .001]
Low job tenure (LJT)	.003	[− .0003, .010]
Difference between HJT and LJT conditions	− .004	[− .011, − .0004]
Perceived overqualification → *work organization *→ needs-supplies fit → task performance		
Indirect effect (M_4_)	− .0001	[− .001, .001]
Moderator employment contract: conditional indirect effects (moderated mediation model, M_5_)		
Temporary contract (TC)	− .001	[− .005, .003]
Permanent contract (PC)	.0001	[− .001, .001]
Difference between TC and PC conditions	− .001	[− .006, .004]
Moderator job tenure: conditional indirect effects (moderated mediation model, M_5_)		
High job tenure (HJT)	− .0001	[− .001, .001]
Low job tenure (LJT)	.0003	[− .002, .003]
Difference between HJT and LJT conditions	.0004	[− .003, .002]

Perceived overqualification → *work organization *→ demands-abilities fit → OCBO		
Indirect effect (M_4_)	.00004	[− .002, .002]
Moderator employment contract: conditional indirect effects (moderated mediation model, M_5_)		
Temporary contract (TC)	.0003	[− .009, .008]
Permanent contract (PC)	− .0001	[− .002, .002]
Difference between TC and PC conditions	.0003	[− .010, .009]
Moderator job tenure: conditional indirect effects (moderated mediation model, M_5_)		
High job tenure (HJT)	.00003	[− .002, .001]
Low job tenure (LJT)	− .0001	[− .004, 005]
Difference between HJT and LJT conditions	.0002	[− .005, .005]
Perceived overqualification → *work organization *→ needs-supplies fit → OCBO		
Indirect effect (M_4_)	− .002	[− .007, .001]
Moderator employment contract: conditional indirect effects (moderated mediation model, M_5_)		
Temporary contract (TC)	− .009	[− .025, − .0004]
Permanent contract (PC)	.002	[− .001, .007.]
Difference between TC and PC conditions	− .011	[− .029, − .001]
Moderator job tenure: conditional indirect effects (moderated mediation model, M_5_)		
High job tenure (HJT)	− .001	[− .005, .001]
Low job tenure (LJT)	.005	[− .0002, .014]
Difference between HJT and LJT conditions	− .006	[− .016, − .0004]
Perceived overqualification → *work organization *→ demands-abilities fit → job satisfaction		
Indirect effect (M_4_)	.0001	[− .001 .002]
Moderator employment contract: conditional indirect effects (moderated mediation model, M_5_)		
Temporary contract (TC)	.001	[− .006, .007]
Permanent contract (PC)	− .0001	[− .002, .002]
Difference between TC and PC conditions	.001	[− .007, .008]
Moderator job tenure: conditional indirect effects (moderated mediation model, M_5_)		
High job tenure (HJT)	.0001	[− .001, .001]
Low job tenure (LJT)	− .0003	[− .003, .003]
Difference between HJT and LJT conditions	.0004	[− .004, .004]
Perceived overqualification → *work organization *→ needs-supplies fit → job satisfaction		
Indirect effect (M_4_)	− .004	[− .012, .001]
Moderator employment contract: conditional indirect effects (moderated mediation model, M_5_)		
Temporary contract (TC)	− .019	[− .047, − .002]
Permanent contract (PC)	.004	[− .003, .013]
Difference between TC and PC conditions	− .023	[− .054, − .004]
Moderator job tenure: conditional indirect effects (moderated mediation model, M_5_)		
High job tenure (HJT)	− .002	[− .010, .004]
Low job tenure (LJT)	.010	[− .0002, .026]
Difference between HJT and LJT conditions	− .012	[− .029, − .002]

Significant conditional indirect effects and differences between conditional indirect effects for permanent vs. temporary contract and high (+ 1 *SD*) vs. low (− 1 *SD*) job tenure are in bold. Employment contract: 0 = permanent contract; 1 = temporary contract

**Fig. 2:**
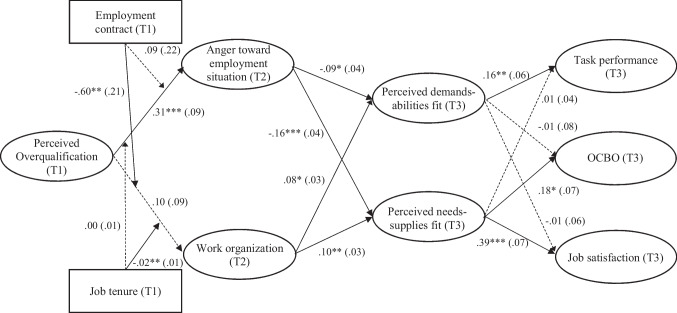
Unstandardized estimated SEM coefficients of the moderated mediation model (M_5_). T1/T2/T3 = first/second/third measurement time point. Effects of age and education on first- and second-stage mediators and outcomes, effects of neuroticism on first-stage mediators, direct effects of moderators on first- and second-stage mediators and outcomes, as well as T1 controls for second-stage mediators and outcomes are not included in the Figure for clarity, but can be found in Table 4. Solid lines are statistically significant, whereas dashed lines are not significant. For employment contract: permanent contract = 0; temporary contract = 1. *p < .05, **p < .01, ***p < .001

**Fig. 3:**
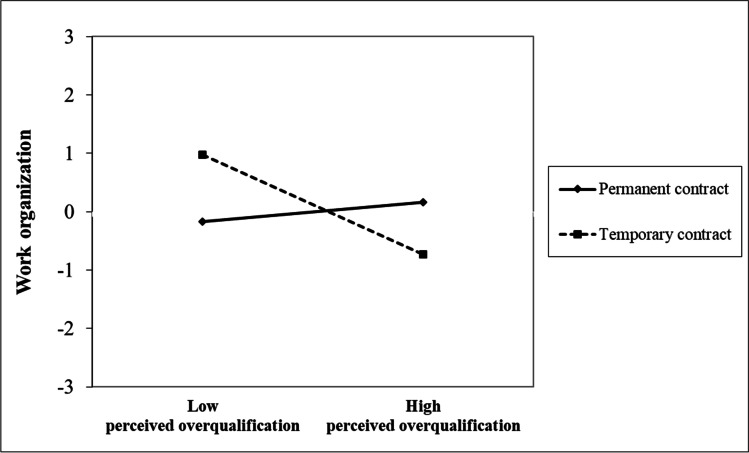
Employment contract moderates the effect of perceived overqualification on work organization

To test for moderation effects in hypotheses 3 and 4, we estimated a moderated mediation model (M_5_, see Fig. 2 and Table 2 for model comparison between M_4_ and M_5_) on the basis of model M_4_ using the numerical integration technique (Klein & Moosbrugger, 2000). This model further included the latent interaction effects of (a) perceived overqualification and employment contract, and (b) perceived overqualification and job tenure on the first stage mediators (i.e., anger and work organization), respectively. Table 4 displays a summary of all model coefficients. For hypothesis 3a, Fig. 2 (see also Table 4) shows that the latent interaction between perceived overqualification and employment contract was not related to anger. Thus, hypothesis 3a was not supported. For hypothesis 3b, Fig. 2 (see also Table 4) shows that the latent interaction between perceived overqualification and employment contract was significantly related to work organization. Fig. 3 graphically displays the interaction effect. Simple slopes tests showed that there was no relationship between perceived overqualification and work organization for employees with a permanent employment contract (γ = 0.101, *p* = 0.276), whereas perceived overqualification was negatively related to work organization for employees with a temporary employment contract (γ =  − 0.503, *p* = 0.013). Fig. 3 clearly shows the demotivating effect of a temporary contract, yet the pattern is different than what we had initially proposed (i.e., positive relationship between perceived overqualification and work organization among employees with a permanent contract, and a less positive relationship between perceived overqualification and work organization among employees with a temporary contract). Hypothesis 3b was thus not supported.

**Table 4:** Unstandardized estimated SEM coefficients of the moderated mediation model (M_5_)

	Anger toward employment situation		Work organization		Demands-abilities fit		Needs-supplies fit		Task performance		OCBO		Job satisfaction	
Variables	Estimate	*SE*	Estimate	*SE*	Estimate	*SE*	Estimate	*SE*	Estimate	*SE*	Estimate	*SE*	Estimate	*SE*
Control variables (T1)														
Age	− .001	.006	− .014*	.007	− .001	.004	.001	.004	.003	.002	.001	.005	.000	.003
Education	.066	.047	.029	.052	− .053	.032	− .027	.030	− .018	.021	.012	.037	− .047	.026
Neuroticism	.386***	.061	− .177**	.065										
Baseline control variables (T1)														
Demands-abilities fit					.631***	.071								
Needs-supplies fit							.592***	.056						
Task performance									.495***	.055				
OCBO											.729***	.048		
Job satisfaction													.542***	.053
Independent variables (T1)														
Perceived overqualification	.306***	.086	.101	.093			− .089**	.031						
Employment contract	− .376	.261	.120	.243	.221	.197	.130	.194	− .091	.124	− .054	.202	.067	.121
Job tenure	− .009	.008	− .001	.011	.016**	.005	.014**	.005	.001	.003	− .010	.006	− .001	.004
Interactions (T1)														
Perceived overqualification × employment contract	.093	.216	− .604**	.211										
Perceived overqualification × job tenure	.000	.005	− .019**	.007										
First-stage mediators (T2)														
Anger toward employment situation					− .094*	.039	− .161***	.041						
Work organization					.083*	.033	.097**	.034						
Second-stage mediators (T3)														
Demands-abilities fit									.161**	.055	− .006	.083	− .014	.062
Needs-supplies fit									.013	.035	.180*	.070	.394***	.071
Residual Variances														
R^2^	30.0%		8.0%		52.7%		66.4%		53.1%		68.8%		83.0%	

*N* = 453. T1/T2/T3 = first/second/third measurement time point. *SE* = standard error. For education: mandatory years of education = 0; vocational training = 1; university entrance diploma = 2; technical college or master craftsman’s diploma = 3; university degree = 4. For employment contract: permanent contract = 0; temporary contract = 1

^*^p < .05; ** *p* < .01; *** *p* < .001

**Fig. 4:**
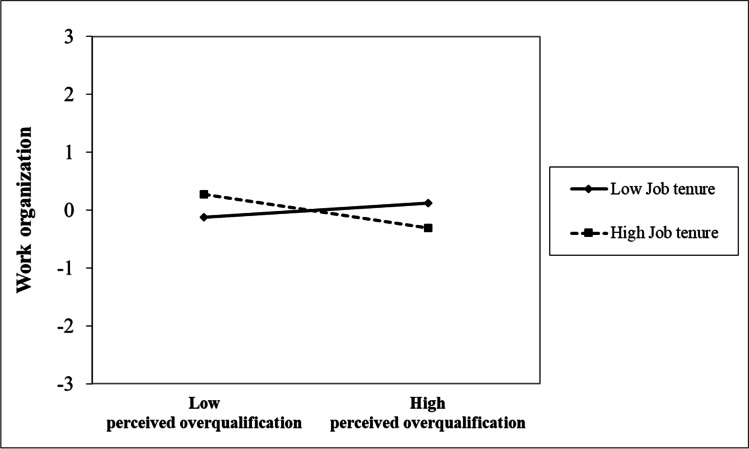
Job tenure moderates the effect of perceived overqualification on work organization

For hypothesis 4a, Fig. 2 (see also Table 4) shows that the latent interaction between perceived overqualification and job tenure was not related to anger. Thus, hypothesis 4a was not supported. For hypothesis 4b, Fig. 2 (see also Table 4) shows that the latent interaction between perceived overqualification and job tenure was significantly related to work organization. Fig. 4 graphically displays the interaction effect. There was no relationship between perceived overqualification and work organization for employees with low job tenure (γ = 0.094, *p* = 0.303), whereas perceived overqualification was negatively related to work organization for employees with high job tenure (γ =  − 0.228, *p* = 0.012). Similar to before, Fig. 4 indicates the demotivating effect of high job tenure, yet shows a different pattern than what we had initially proposed (i.e., positive relationship between perceived overqualification and work organization among employees with low job tenure, and a less positive relationship between perceived overqualification and work organization among employees with high job tenure). Hypothesis 4b was thus not supported.

Further, although not hypothesized, we estimated indirect effects at conditional values of the moderator (i.e., permanent vs. temporary employment contract and 1 *SD* above and below the mean of job tenure) and corresponding differences as displayed in Table 3. Results showed a significant difference between values of conditional indirect effects for permanent vs. temporary employment contract for the relationship between perceived overqualification and changes in task performance through work organization (i.e., first-stage mediator) and perceived demands-abilities fit (i.e., second-stage mediator), for the relationship between perceived overqualification and changes in OCBO through work organization and perceived needs-supplies fit, and for the relationship between perceived overqualification and changes in job satisfaction through work organization and perceived needs-supplies fit. Moreover, there was a significant difference between values of conditional indirect effects for low vs. high tenure for the relationship between perceived overqualification and changes in task performance through work organization and perceived demands-abilities fit, as well as for the relationships between perceived overqualification and changes in OCBO and job satisfaction through work organization and perceived needs-supplies fit.

### Supplemental Analyses

We conducted a series of supplemental analyses in order to examine the robustness of our findings. First, to estimate the impact of our control variables, we followed recommendations on the use of control variables (e.g., Spector & Brannick, 2011) and additionally tested our hypotheses without control variables. Results were the same as in the model without controls, with the exception that perceived overqualification was negatively related to work organization in the full mediation model (M_4_). This effect became nonsignificant when entering the interactions in the moderated mediation model (M_5_). Full analysis tables can be requested from the corresponding author.

Second, we conducted a set of sensitivity analyses due to the skewed distribution of the tenure variable (skewness = 1.56, kurtosis = 2.44). To do so, we followed recommendations by Aguinis et al. (2013) and conducted an outlier analysis whereby we re-ran our model after excluding (a) participants who indicated job tenure values in the bottom and top 2.5% of the tenure distribution (resulting in a sample size of *N* = 425), (b) respondents with a tenure that was below or above 2.24 standard deviations (resulting in a sample size of *N* = 434). Moreover, we applied a more conservative rule (Darlington & Hayes, 2016; Kline, 2011) and also (c) re-ran our model after excluding respondents with a tenure that was below or above 2 standard deviations of the tenure mean (resulting in a sample size of *N* = 428). Finally, we (d) log-transformed the tenure variable (Aguinis et al., 2013). In short, all results remained unchanged. More detailed results can be requested from the corresponding author. Taken together, results from our supplemental analyses underscore the robustness of our findings.

## Discussion

Rooted in the tenets of P-E fit theory, we united two types of adjustment reactions—a strain-based and a self-regulatory pathway—that are elicited by perceived overqualification, along with indicators of formal work arrangements as their boundary conditions. First, and in line with our assumptions, we found that perceived overqualification was indirectly related to a decrease in task performance, OCBO, and job satisfaction, respectively, via higher levels of anger toward employment situation in the first step and lower levels of perceived demands-abilities fit and perceived needs-supplies fit, respectively, in the second step. Second, we found that type of contract and job tenure moderated the relationship between perceived overqualification and work organization. The pattern of interactions, however, was different from what we had initially expected. Among employees with a temporary contract or with longer job tenure, the relationship between perceived overqualification and work organization was negative and significant; this relationship was non-significant among employees with a permanent contract or with short job tenure.

### Theoretical Implications

The current study yields several theoretical implications. First, our findings point to the importance of viewing situations of misfit, such as perceived overqualification in the present case, from a more integrative perspective in line with P-E fit theory. As noted in the introduction, previous research on perceived overqualification has largely focused on the strain-based pathway and related outcomes. While there is some preliminary evidence on self-regulatory behaviors, these studies have theoretically treated such behaviors in isolation (e.g., Zhang et al., 2016). Our research points to the notion that self-regulatory adjustment reactions are likewise rooted in the tenets of P-E fit theory. In the present research, we therefore explicitly united both pathways in the context of perceived overqualification, thus achieving a more integrated understanding of this phenomenon and its elicited processes. Albeit the results for the two moderators in the context of work organization did not support our initial hypotheses, we nevertheless believe that the pattern of findings makes a meaningful contribution to the literature in that it highlights the particularly demotivating role that a temporary contract and long job tenure can have—thus also bearing important practical implications (see below for a more detailed discussion).

Second, by examining the mediational effects of person-job fit perceptions, we contribute to investigating the process underlying the effects of the strain- and self-regulatory pathways on job performance and job satisfaction in line with P-E fit theory (Edwards et al., 1998; Follmer et al., 2018; Yu, 2009). More precisely, we examined how strain and self-regulatory behavior resulting from perceived overqualification provide feedback to perceived person-job fit. In doing so, we contribute to examining more dynamic features of P-E fit theory—an issue that has been rarely explicitly tested in previous research, yet been called for (e.g., Yu, 2013).

Third, by examining the moderating roles of type of contract and job tenure as indicators of formal work arrangements, our study shows that adjustment reactions in line with P-E fit theory are affected by contextual boundary conditions. Our findings emphasize that the self-regulatory pathway, in particular, is not necessarily elicited by perceived overqualification in a uniform way; instead, such reactions are further shaped by the contextual complexities that employees are embedded in. In addition, by demonstrating the moderating role of type of contract and job tenure, we identify two key boundary conditions that have gained considerable attention in different literatures (e.g., De Cuyper et al., 2009; Ng & Feldman, 2013). We thus extend the spectrum of theoretically meaningful boundary conditions that researchers and practitioners need to consider when dealing with the phenomenon of perceived overqualification, thus helping to answer the question “of when, and for whom, it [i.e., perceived overqualification] is problematic” (Simon et al., 2019, p. 214).

Some findings merit further consideration. First, and as alluded to before, the general pattern of moderating effects for work organization did not align with our propositions. Whereas the relationship between perceived overqualification and work organization was non-significant for employees on a permanent contract, the relationship was significantly negative for those with a temporary contract. It thus appears that having a temporary contract is an even stronger demotivating force than we had initially assumed. Because perceived overqualification is coupled with an insecure and unstable work contract situation (e.g., Kalleberg, 2009), employees appear to experience a double-whammy effect that makes them refrain from all self-regulatory efforts. For job tenure, a similar finding emerged. For employees with shorter job tenure, the relationship between perceived overqualification and work organization was nonsignificant, but it became negative for employees with longer job tenure. Bearing in mind the losses in motivation among employees with longer job tenure discussed earlier (e.g., Ng & Feldman, 2013), these findings highlight the importance of considering the role of job stages and motivational processes in the context of perceived overqualification.

Second, our analyses did not reveal any interaction effects between perceived overqualification and the two indicators of formal work arrangements when predicting anger toward employment situation as reflecting the strain-based pathway. One potential explanation might be that the strain-based reaction to perceived overqualification is quite strong, thus overshadowing any contingency effect. Finally, we found that perceived demands-abilities fit was primarily predictive of an increase in task performance, whereas perceived needs-supplies fit was primarily predictive of an increase in OCBO and job satisfaction. Although we had originally assumed that both types of fit should be equally important for all three outcomes, perceived demands-abilities fit might be more relevant for task performance due to its focus on skills-based task proficiency (for a similar argument see Kristof-Brown et al., 2005). In contrast, perceived needs-supplies fit might be more relevant for OCBO due to processes related to social exchange (Blau, 1964). More precisely, organizational commitment, which is among the primary drivers of OCB (LePine et al., 2002), has been shown to be more strongly affected by needs-supplies fit as compared to demands-abilities fit (Kristof-Brown et al., 2005). Similarly, needs-supplies fit might be more relevant for job satisfaction due to the aforementioned comparison processes involved. Indeed, Edwards and Shipp (2007) noted that “subjective needs-supplies fit parallels the comparison process underlying theories of job satisfaction” (p. 221), thus rendering it a more proximal predictor of job satisfaction. The authors also noted that demands-abilities fit might be a more distal predictor of job satisfaction, thus alluding to potentially mediating mechanisms that we also referred to in our hypothesis development.

Finally, based on suggestions in the review process, we examined a set of further models with alternative configurations of our study variables, thus testing potentially alternative mechanisms. First, we examined whether work organization moderates the relationship between perceived overqualification and anger (while keeping the rest of the model as is). This was not the case. Second, we examined a three-mediator chain model whereby perceived overqualification relates to anger, which in turn relates to work organization (while keeping the rest of the model as is). Whereas perceived overqualification was negatively related to anger (as in our main model), anger was not found to relate to work organization. Third, we examined whether neuroticism moderates the relationship between perceived overqualifications and anger and work organization, respectively. None of the interactions yielded a significant effect. In sum, the non-significant findings from these alternative models further underline the robustness of our model, thus underscoring the soundness of our initially proposed dual-pathway model.

### Practical Implications

Our findings also yield practical implications for managers and organizations in terms of dealing with overqualified employees. First, our findings highlight the detrimental effects of temporary employment contracts in the context of perceived overqualification. As discussed above, having a temporary contract had a stronger demotivating effect on self-regulatory behavior in the form of work organization than we had initially assumed. Although the general precarity and instability associated with a temporary contract has been heavily discussed and documented in the literature (e.g., Kalleberg, 2000, 2009), numbers tend to be on the rise worldwide (OECD, 2021). Given the fact that numbers of overqualified employees show a somewhat similar trend (e.g., McGuiness et al., 2018), we strongly advocate for organizations to avoid such contracts whenever possible. If this is not possible in certain businesses (e.g., seasonal work), organizations should at least communicate openly to their employees about what will happen when the contract ends (e.g., Snyder & Morris, 1984) and/or offer programs to support successful job transitions.

Second, our findings also point to the demotivating effect of long job tenure for engaging in work organization among overqualified employees. Since more tenured employees are experienced job performers with a great deal of knowledge about organizational processes and structures, organizations and managers need to be attentive to them. In a related vein, organizations may become more open towards the mindset of hiring (overqualified) employees for the organization instead of hiring them for a particular job (Kulkarni et al., 2015). By providing developmental pathways within the organization and/or job rotation opportunities, employees might uphold high levels of work motivation and potentially directly reduce their perceived overqualification. Likewise, in doing so organizations would be better able to capitalize on the manifold skills and abilities that overqualified employees bring with them (Martinez et al., 2014).

Finally, our findings revealed a consistent strain-based pathway of perceived overqualification via higher levels of anger toward employment situation, thus corroborating previous research (e.g., Harari et al., 2017). Because strain reactions can elicit further detrimental outcomes (Sonnentag & Frese, 2012), managers need to be aware if their employees perceive themselves to be overqualified. We thus advise managers to engage in regular meetings with their employees in order to gauge whether or not perceived overqualification is an issue. For example, if overqualification is an issue, managers may then assign these employees more autonomy (Debus et al., 2020; Erdogan & Bauer, 2009) and/or provide developmental pathways within the organization (Kulkarni et al., 2015).

### Limitations and Directions for Future Research

Our study is not without limitations. First, our findings were based solely on self-report data. Thus, it remains open whether the same pattern of findings would have emerged if some measures had been collected from a different source, such as by employing supervisor ratings of performance. Yet, to avoid problems related to common source data, we temporally separated the measurements (Podsakoff et al., 2012). Moreover, we controlled for effects of neuroticism on both anger and work organization, as well as the respective baseline levels of perceived person-job fit and the performance measures, thus achieving an even more robust test of our assumptions.

Second, the current research focused on two indicators of formal work arrangements (i.e., employment contract and job tenure) and demonstrated their impactful role for effects of perceived overqualification on work organization. Yet, there are other work arrangements that may play a role in the present context. For example, the opportunity to work remotely^3^ may act as a further moderator in our model—and there are two effects that appear plausible. On the one hand, the opportunity to work remotely may be perceived as a great benefit and as a type of autonomy (Gajendran & Harrison, 2007) that may spur feelings of organizational support (Eisenberger et al., 1986). Overqualified employees may thus be more motivated to change their jobs for the better, leading to higher levels of self-regulatory behavior. On the other hand, working remotely may also contribute to the deterioration in employees’ interactions with their coworkers and supervisors (Gajendran & Harrison, 2007). This, in turn, might lower their commitment and attachment to their organization, such that overqualified employees may lose the motivation to engage in self-regulatory behavior. We encourage future research to examine these effects in more detail, as well as to expand more generally upon the interplay of perceived overqualification and formal work arrangements.

Third, future research may examine whether our findings can be generalized to further types of outcomes. Albeit we focused on positive key performance and well-being indicators, earlier research has demonstrated that counterproductive work behaviors (CWB) constitute a prevalent outcome of perceived overqualification (Harari et al., 2017), and that anger constitutes one of the main mechanisms through which CWB is elicited (Liu et al., 2015; Shockley et al., 2012). Likewise, overqualified employees may attribute their situation more specifically to their immediate manager as being the person in charge for their situation and the development of their career. It is thus conceivable that effects may generalize to a specific interpersonal form of OCB that is directed at the supervisor (Bentein et al., 2002).

Fourth, our conceptualization of perceived overqualification in this study aligned with earlier research in the field that considers the phenomenon as a relatively enduring job stressor at the level of the entire job (e.g., Erdogan & Bauer, 2021; Harari et al., 2017). For this reason, we also focused on anger (toward employment situation) as a mood state as a somewhat longer-lasting reaction. Yet, as mentioned in the theory section, anger can also be experienced as a short-lived, high intensity emotional reaction. By conceptualizing perceived overqualification on a state level, such as in the form of a daily phenomenon relating to specific tasks that one is dealing with on a specific day, future research may examine the degree of homology of effects across different levels of analysis (Kozlowski & Klein, 2000). Put differently, it is conceivable that if employees experience relatively higher levels of task-related overqualification on a specific day (compared to how they on average experience the nature of their tasks), this may elicit relatively higher levels of short-lived anger emotions on that day, further impacting daily perceptions of person-job fit, as well as daily performance and job satisfaction levels.

Finally, as overqualification is a labor market occurrence, and thus, an economic phenomenon by nature, future research may apply a multilevel perspective that more explicitly considers the macro-economic context for understanding why employees become overqualified as well as how they react to it. As an example, certain industries worldwide currently face an oversupply of workers (e.g., commercial and administrative sectors, as well as cleaning, and hygiene) which leads to great competition among jobseekers (e.g., Aims Global, 2021; Spring Professional, 2020). Due to most individuals’ pressure to earn wages and to avoid unemployment, jobseekers in these sectors may be more likely to end up in an employment situation in which they feel overqualified. In other words, being in a context with limited economic alternatives may make individuals more likely to tolerate a job with less than optimal fit. In a similar vein, it is conceivable that in times of high (regional or country-level) unemployment, employees are more willing to tolerate a job that is below their qualifications because they are aware of the high competition on the labor market (e.g., Carsten & Spector, 1987). Whereas the management and psychology literature has traditionally focused on individual (e.g., demographic, personality and job-related) antecedents and moderators of perceived overqualification (Erdogan & Bauer, 2021), applying a multilevel perspective that explicitly connects the (macro)economic context with the individual level will contribute to a more holistic understanding of the construct and its related processes (e.g., Sinclair et al., 2010). We thus strongly encourage research into this direction.

In a related vein, some readers may be surprised regarding the relatively high average job tenure (8.78 years) in our sample. As noted in the method section, our data were collected in Germany, a country with strong social protection of workers [e.g., laws related to dismissal protection (e.g., Eurofound, 2021), as well as a considerable number of employees being trade union members (International Labour Organization, 2022)]. Indeed, official statistics demonstrate that in 2020 average tenure among German workers was above 10 years (OECD, 2022a; Statistisches Bundesamt, 2022)—an aspect that can be likewise observed in many other European countries (OECD, 2022a). The tenure distribution in our sample thus well reflects official population statistics.

### Conclusion

The overarching goal of this study was to unite two qualitatively different adjustment reactions to perceived overqualification based on P-E fit theory. We demonstrated that perceived overqualification concurrently elicits strain-based and self-regulatory reactions, albeit in a somewhat different manner than we had initially proposed. Moreover, this study demonstrates the important role of formal work arrangements for further shaping the self-regulatory pathway. Taken together, this study contributes to a more integrative understanding of perceived overqualification and its underlying processes from the perspective of P-E fit, thereby also pointing to the impact different contract types and different job stages have on motivation and performance.

## Data Availability

The data that support the findings of this study are available from the corresponding author, upon reasonable request.

## Footnotes

1 For further information on this company, see www.respondi.com.

2 More detailed information about sample comparisons can be obtained from the corresponding author.

3 We had originally planned to examine remote work as an additional boundary condition in this study. Yet, during the course of our data collection the German COVID-19 Occupational Safety and Health Ordinance was put in place. German employers were thus mandated by law to offer their employees the opportunity to work remotely whenever possible. Because the opportunity to work remotely became prescribed by the government, the concept itself became different from a formal work arrangement. We thus no longer considered the opportunity to work remotely as a formal work arrangement in this study.
